# Ultrafast and low-energy switching in voltage-controlled elliptical pMTJ

**DOI:** 10.1038/s41598-017-16292-7

**Published:** 2017-11-29

**Authors:** Jiefang Deng, Gengchiau Liang, Gaurav Gupta

**Affiliations:** 1 Electrical and Computer Engineering, National University of Singapore, 117576 Singapore, Singapore; 2 Centre for Advanced 2D Materials and Graphene Research Centre, National University of Singapore, Singapore, 117546 Singapore; 3 Spin Devices, Delhi, 110006 India

## Abstract

Switching magnetization in a perpendicular magnetic tunnel junction (pMTJ) via voltage controlled magnetic anisotropy (VCMA) has shown the potential to markedly reduce switching energy. However, the requirement of an external magnetic field poses a critical bottleneck for its practical applications. In this work, we propose an elliptical-shaped pMTJ to eliminate the requirement of providing an external field by an additional circuit. We demonstrate that a 10 nm thick in-plane magnetized bias layer (BL) separated by a metallic spacer of 3 nm from the free layer (FL) can be engineered within the MTJ stack to provide the 50 mT bias magnetic field for switching. By conducting macrospin simulation, we find that a fast switching in 0.38 ns with energy consumption as low as 0.3 fJ at a voltage of 1.6 V can be achieved. Furthermore, we study the phase diagram of switching probability, showing that a pulse duration margin of 0.15 ns is obtained and low-voltage operation (~1 V) is favored. Finally, the MTJ scalability is considered, and it is found that scaling down may not be appealing in terms of both the energy consumption and the switching time for precession based VCMA switching.

## Introduction

Spin transfer torque (STT) based magnetic random access memory (MRAM)^1,2^ because of its non-volatility, high access speed and CMOS (complementary metal oxide semiconductor) compatibility^3^ has matured into one of a leading candidate in recent years^4^ to fill memory gaps in the extant memory hierarchy. A bit-cell of STT-RAM comprises of a magnetic tunnel junction (MTJ) which has a pinned-layer (PL) and a free-layer (FL) with their magnetization in either parallel (P) or anti-parallel (AP) state with respect to (w.r.t.) each other, which corresponds to logic “1” or “0”. In STT-RAM, a free-layer is written by passing a current with density larger than critical current density through the MTJ. If the current is flowing from FL towards PL, a spin-flux with vector parallel to the magnetization of PL (**M**_**PL**_) acts on the FL to align the magnetization of FL (**M**_**FL**_) with **M**_**PL**_, whereas if the current direction is reversed a reverse spin-flux with vector anti-parallel to **M**_**PL**_ acts on the FL to orient it in the AP state. This current based magnetization switching, however, requires a large current density which ranges from 5 × 10^10^ A-m^−2^ for 35 ns to 1 × 10^11^ A-m^−2^ for 2 ns write-time^1,2^ to generate enough spins to toggle all magnetic moments of the FL. Inevitably, a large current results in considerable Joule heating in the MTJ. This results in self-heating^5–7^ induced degradation of the MTJ characteristics, e.g. the spin-polarization of spin-flux degrades thereby degrading the STT efficiency at higher temperatures. In addition, electromigration^8,9^ becomes prominent because of large current densities, and the dielectric may also break^10^ at voltages required to sustain required current densities. Furthermore, to provide a large enough current a bulky access transistor, i.e. with a large channel width-to-length ratio for bulk-MOSFET or with a large number of fins for FinFET (Fin Field Effect Transistor), is required. This implies that STT-RAM suffers from high energy consumption, reliability issues and a huge cell area^11,12^.

To reduce the operating current which would subsequently reduce both the energy consumption and the size of driving transistor, voltage control of magnetic anisotropy (VCMA)^13^ has been promulgated as an alternative to the STT^14,15^. There are several possible physical origins for VCMA effect. Among them, the redox reaction and the electromigration can result in a VCMA efficiency of 1000 fJ-V^−1^-m^−1^ ^16^. However, the low reacting speed makes it unfeasible for memory application. Therefore, to design a memory cell, the most possible mechanism for VCMA effect is that electric field modulates the charge occupancy at the interface. First principles studies have shown that the modification of magnetic anisotropy by an electric field is contributed to the change of 3d-orbitals occupancies via spin-orbit interaction^17^. Since the VCMA based device relies on a voltage rather than a current to write, the current in this case can be greatly reduced by designing a MTJ with large enough resistance. Furthermore, the VCMA based precessional switching enables the FL to toggle in sub-nanosecond^13,18^. Therefore, the energy consumption can be substantially reduced by markedly reducing both the power-dissipation and the dissipation-time. Demonstrations^18–20^ hitherto have been on circular pMTJs. These demonstrations require an external in-plane magnetic field to enable the switching. This, however, is not a viable solution for integrated MRAM w.r.t. both the provision of an external field source and the field uniformity^21,22^. Consequently, the requirement of an external bias field poses a critical bottleneck for realizing practical VCMA memory.

In this work, therefore, we propose an elliptical pMTJ to eliminate the requirement of an external magnetic field source. Because of the elliptical structure, via shape anisotropy, an in-plane magnetized bias-layer (BL) separated from the FL by a metallic spacer can be directly engineered within the MTJ stack^23^. This in-plane BL, hence, provides a sufficient bias field for VCMA based precessional switching. We comprehensively appraise the effects of electronic, magnetic and physical design constraints on the FL switching dynamics to expound the device physics and an optimal operation window in the proposed design. Our results show that the required bias magnetic field can be contrived within the MTJ stack by an in-plane magnetized BL. For instance, a 10 nm thick BL separated by a 3 nm thick metallic spacer can provide an in-plane exchange field of 50 mT to bias the FL. Furthermore, we show that the FL can toggle in just 0.38 ns consuming only 0.3 fJ at a voltage of 1.6 V across the MTJ, which is attractive for memory applications. Our results also indicate that the pMTJ driven by precession based VCMA favors a low-voltage operation (~1 V) with a sufficient margin (0.15 ns) for the applied voltage pulse duration.

## Methods

**Figure 1:**
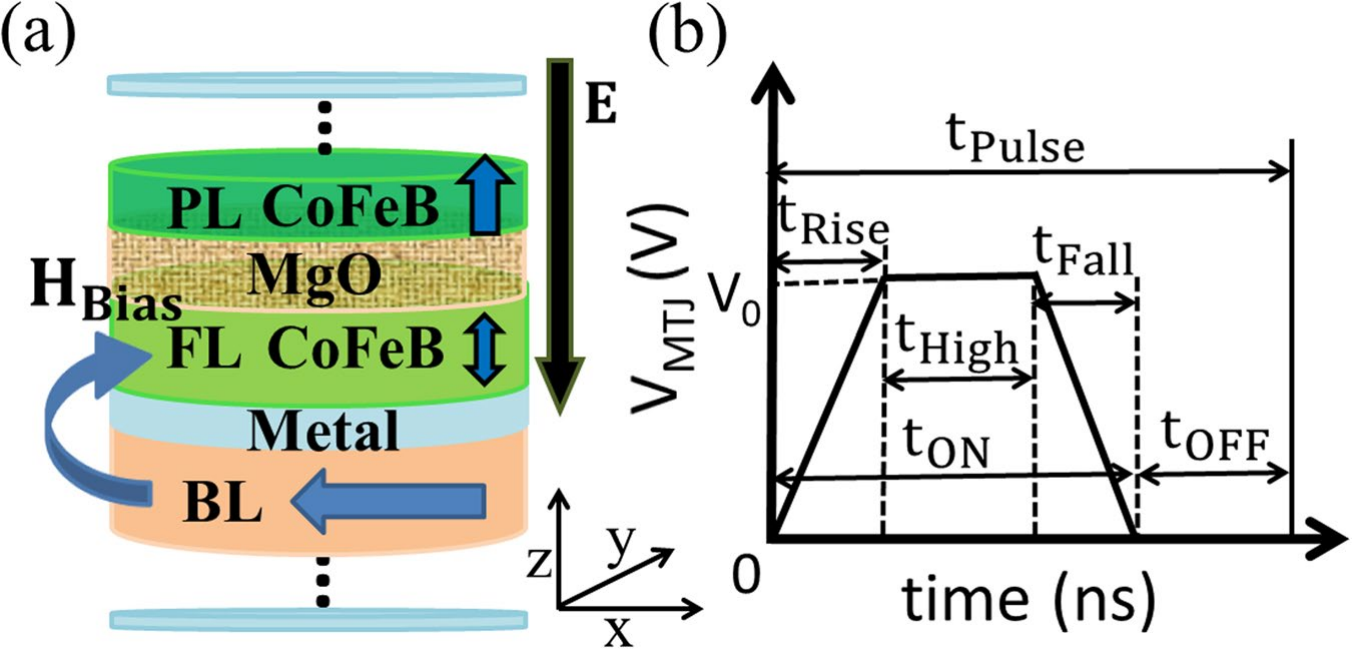
(**a**) Schematic of an elliptical pMTJ with the major axis along the x-axis. A bias layer (BL) separated by a metal is engineered to provide the exchange field for the FL. The top and bottom water-blue layers are electrodes. (**b**) Applied voltage pulse with t_Rise_ = t_Fall_ = 50 ps and t_OFF_ of 10 ns, which is long enough to ensure that the magnetization is completely relaxed. Positive voltage is defined for the electric field pointing from the PL to the FL.

Figure 1(a) shows a schematic of an elliptical MTJ with perpendicular magnetic anisotropy (PMA), with major-axis (minor-axis) along x-axis (y-axis). A 1.2 nm thick Co_20_Fe_60_B_20_^24^ PL and a 1.7 nm thick Co_20_Fe_60_B_20_ FL sandwich a MgO insulator of thickness t_MgO_. In this work, t_MgO_ ranges from 1.2 nm to 2.8 nm, while the MTJ cross-section ranges from 150 × 50 nm^2^ to 114 × 38 nm^2^ with a fixed aspect-ratio (AR) of 3. Macrospin simulation, which has been shown to be valid in purview of dimensions considered in this work^13,18,25,26^, is developed to investigate the magnetization dynamics. The dynamics is described by the Landau-Lifshitz-Gilbert (LLG) equation^27,28^ as,

1 $$\begin{array}{l}\frac{\partial {\bf{m}}}{\partial t}=-\gamma {\bf{m}}\times {\mu }_{0}{{\bf{H}}}_{{\bf{E}}{\bf{f}}{\bf{f}}}+\alpha {\bf{m}}\times \frac{\partial {\bf{m}}}{\partial t}+{{\rm{\Gamma }}}_{\mathrm{MTJ}\_\mathrm{DL}}{\bf{m}}\times {{\bf{p}}}_{{\bf{P}}{\bf{L}}}\times {\bf{m}}+{{\rm{\Gamma }}}_{{\rm{SV}}}{\bf{m}}\times {{\bf{p}}}_{{\bf{B}}{\bf{L}}}\times {\bf{m}}+{{\rm{\Gamma }}}_{\mathrm{MTJ}\_\mathrm{FL}}{\bf{m}}\times {{\bf{p}}}_{{\bf{P}}{\bf{L}}},\end{array}$$

where *γ* is the gyromagnetic ratio, *μ*_0_ is the vacuum permeability, *α* = 0.01^28^ is the Gilbert damping coefficient for Co_20_Fe_60_B_20_, and **p**_**PL**_ (**p**_**BL**_) is a unit-vector anti-parallel (parallel) to the magnetization of PL (BL). The magnetization unit-vector of FL **m** is [m_x_ m_y_ m_z_], which is [0 0 ± 1] in stable states, with m_x_, m_y_ and m_z_ being the projections on respective axis. **H**_**Eff**_ is the effective magnetic field experienced by the FL. It is the vector sum of uniaxial anisotropy field **H**_**K**_, demagnetizing field **H**_**D**_, thermal fluctuation field **H**_**Therm**_, and external bias field **H**_**Bias**_, which are expressed as,

2 $$\begin{array}{l}{{\bf{H}}}_{{\bf{K}}}=\frac{{{\rm{2K}}}_{{\rm{U}}}}{{\mu }_{{\rm{0}}}{{\rm{M}}}_{{\rm{S}}}}[{0,0,m}_{{\rm{z}}}],{{\rm{K}}}_{{\rm{U}}}={{\rm{K}}}_{{\rm{U}}\_\mathrm{Bulk}}+\frac{{{\rm{K}}}_{{\rm{I0}}}-{\rm{\xi }}\cdot {{\rm{E}}}_{{\rm{z}}}}{{{\rm{t}}}_{{\rm{FL}}}},\end{array}$$

3 $$\begin{array}{l}{{\bf{H}}}_{{\bf{D}}}=-{{\rm{M}}}_{{\rm{S}}}[{{\rm{N}}}_{{\rm{x}}}{{\rm{m}}}_{{\rm{x}}},{{\rm{N}}}_{{\rm{y}}}{{\rm{m}}}_{{\rm{y}}},{{\rm{N}}}_{{\rm{z}}}{{\rm{m}}}_{{\rm{z}}}],\end{array}$$

4 $$\begin{array}{l}{{\bf{H}}}_{{\bf{T}}{\bf{h}}{\bf{e}}{\bf{r}}{\bf{m}}}=\sqrt{\frac{2{\rm{\alpha }}{{\rm{K}}}_{{\rm{B}}}{\rm{T}}}{({\rm{1}}+{{\rm{\alpha }}}^{{\rm{2}}}){{\rm{\gamma }}{\rm{M}}}_{{\rm{S}}}{\rm{V}}({\rm{\Delta }}{\rm{t}})}}\frac{{\rm{1}}}{{{\mu }}_{{\rm{0}}}}[{{\rm{G}}}_{(0,1)}^{{\rm{x}}},{{\rm{G}}}_{(0,1)}^{{\rm{y}}},{{\rm{G}}}_{(0,1)}^{{\rm{z}}}],\end{array}$$

where K_U_ is the anisotropy energy density with contributions from bulk anisotropy, K_U_Bulk_, and interfacial anisotropy K_I_. The latter is computed as K_I0_ − ξ ⋅ E_z_. As implied from equation (2), the interfacial anisotropy is assumed to be linearly modified by the perpendicular component E_z_ of the electric-field **E** at the MgO-FL interface, at a rate determined by the VCMA coefficient *ξ* ^24,29^, where equation (2) assumes a positive value of E_z_ for the field direction shown in Fig. 1(a). For the voltage applied across the MTJ V_MTJ_, the magnitude of E_z_ is assumed to be V_MTJ_/t_MgO_^18,20^. The CoFeB and the CoFeB/MgO interface parameters like saturated magnetization M_S_ = 1.257 × 10^6^ A-m^−1^, K_U_Bulk_ = 2.245 × 10^5^ J-m^−3^, K_I0_ = 1.286 × 10^−3^ J-m^−2^ ^24^ and ξ = 50 fJ-V^−1^-m^−1^ ^30^ are empirical parameters from experimental papers. The value of *ξ* at the CoFeB/MgO interface in literature is in the range of 20–100 fJ-V^−1^-m^−1^ ^30–33^. More efficient VCMA effect, i.e. larger *ξ*, would result in even better performance of the proposed device than that predicted in this work.

In equation (3), N_x_, N_y_ and N_z_ are the demagnetizing factors along x, y and z directions, which are determined by the shape and the size of magnet (shape anisotropy)^34,35^. The dipole field from the PL has been neglected assuming that this dipole field can be cancelled out by synthetic ferrimagnetic reference layers^36^. Thermal fluctuation is described by equation (4), where K_B_, V and Δt are the Boltzmann constant, the FL volume and the calculation time step of 5 ps, respectively. The stochastic partial differential equation (SPDE) described by equation (1) is integrated via fourth order Runge-Kutta method^37–39^. The device is assumed to operate at room temperature (T = 300 K), and self-heating effects due to Joule heating have been ignored because the VCMA devices operate at much lower current densities than traditional STT devices. G_(1,0)_ with superscript along respective axis are independent random numbers computed at every time-step and each has a Gaussian distribution with zero mean and unit standard deviation^40^. **H**_**Bias**_ (c.f. Fig. 1(a)) is provided by an in-plane magnetized BL (Co/Pt multilayers) via its dipole field, which is calculated by micromagnetics simulation using MuMax3 simulator^41^ with the simulation cell size of 1 nm along all three dimensions. Since **H**_**Bias**_ is along x-axis in this study, subsequently, it is represented by H_x_. The damping-like torque (DLT) with linear dependence on V_MTJ_, and the field-like torque (FLT) with quadratic dependence on V_MTJ_ are respectively obtained as,

5 $$\begin{array}{l}{{\rm{\Gamma }}}_{\mathrm{MTJ}\_\mathrm{DLT}(\mathrm{SV})}=\frac{{\rm{\hbar}}}{{\rm{2e}}}\frac{{\rm{\gamma }}{{\eta }}_{\mathrm{MTJ}(\mathrm{SV})}}{{{\rm{M}}}_{{\rm{S}}}{{\rm{VR}}}_{{\rm{MTJ}}}}{{\rm{V}}}_{{\rm{MTJ}}},\quad {{\rm{\Gamma }}}_{\mathrm{MTJ}\_\mathrm{FLT}}={\rm{\nu }}\frac{\rm{\hbar}}{{\rm{2e}}}\frac{{\rm{\gamma }}{{\eta }}_{{\rm{MTJ}}}}{{{\rm{M}}}_{{\rm{S}}}{{\rm{VR}}}_{{\rm{MTJ}}}}{{\rm{V}}}_{{\rm{MTJ}}}^{{\rm{2}}},\end{array}$$

where $\hslash$ is the reduced Planck constant, *e* is the electron charge, R_MTJ_ is the MTJ resistance, η_MTJ(SV)_ is the STT efficiency, and *ν* = 2.97/7.82 V^−1^ is the ratio between the two torques^42^. Analysis of R_MTJ_ includes the voltage dependence of tunneling magnetoresistance (TMR) and the dynamic angle *θ* between FL and PL as,

6 $$\begin{array}{l}{{\rm{R}}}_{{\rm{MTJ}}}={{\rm{R}}}_{{\rm{P}}}+\frac{{{\rm{R}}}_{{\rm{AP0}}}-{{\rm{R}}}_{{\rm{P}}}}{{\rm{1}}+\frac{{{\rm{V}}}_{{\rm{MTJ}}}^{{\rm{2}}}}{{{\rm{V}}}_{{\rm{Half}}}^{{\rm{2}}}}}(\frac{{\rm{1}}-\,\cos ({\theta })}{{\rm{2}}}),\end{array}$$

where V_Half_ = 0.4 V^20,43^, is the voltage across MTJ at which TMR becomes half of its value at zero-bias i.e. TMR_0_/2. R_P_ is the MTJ resistance when both magnets are exactly parallel to each other and assumed to remain invariant to V_MTJ_^42,44^, while R_AP0_ is the MTJ resistance when the MTJ is in AP state at zero bias. Since in recent years Slonczewski expression^27^ for spin-torque efficiency has been extended to account for multiple reflections of the spin-flux in spin valves^45–47^, the STT effect by the BL in this study is based on multi-reflection model. Hence, the STT efficiencies for the PL-MgO-FL MTJ (η_MTJ_) and the FL-Metal-BL spin valve (η_SV_) are computed as^46,48^,

7 $$\begin{array}{l}\begin{array}{c}{{\eta }}_{{\rm{MTJ}}}=\frac{{{\rm{P}}}_{{\rm{1}}}}{{\rm{1}}+{{\rm{P}}}_{{\rm{1}}}^{{\rm{2}}}\,\cos ({\theta })},\\ \,\,\,{{\rm{P}}}_{{\rm{1}}}=\sqrt{\frac{{{\rm{TMR}}}_{{\rm{0}}}/2}{{\rm{1}}+{{\rm{TMR}}}_{{\rm{0}}}/2}},\\ {{\eta }}_{{\rm{SV}}}=\frac{{{\rm{P}}}_{{\rm{2}}}-{{\rm{P}}}_{{\rm{2}}}{\rm{\zeta }}\,\cos ({\theta })}{{\rm{1}}-{{\rm{\zeta }}}^{{\rm{2}}}{\cos }^{{\rm{2}}}({\theta })},\\ \,\,{\rm{\zeta }}={\rm{1}}-2{\rm{\varepsilon }}+{2{\rm{\varepsilon }}}^{{\rm{2}}},\\ \,\,\,\,{\rm{\varepsilon }}=\frac{{\rm{1}}-{{\rm{P}}}_{{\rm{2}}}}{{\rm{2}}},\end{array}\end{array}$$

where P_1_ is obtained from Julliere’s formula for equal polarization of FL and PL^49^ and P_2_ = 0.35^50^.

### Data Availability

Correspondence and requests for materials should be addressed to G.G. (gauravdce07@gmail.com) or G.L. (elelg@nus.edu.sg).

## Results and Discussion

### Operation Principle

Since in a VCMA based MTJ, the interfacial anisotropy energy can be tuned by an applied voltage, the competition between the uniaxial anisotropy and the demagnetizing field, which determines the easy axis direction, can be controlled by voltage. Specifically, considering the bulk and the interface PMA with the demagnetization simultaneously, the net anisotropy energy density becomes,

8 $$\begin{array}{l}{{\rm{K}}}_{{\rm{U}}\_{\rm{Eff}}}\,=\,{{\rm{K}}}_{{\rm{U}}}-\frac{{\rm{1}}}{{\rm{2}}}{{\mu }}_{{\rm{0}}}{{\rm{M}}}_{{\rm{S}}}^{{\rm{2}}}{{\rm{N}}}_{{\rm{z}}}\mathrm{.}\end{array}$$

When K_U_Eff_ > 0, FL has a perpendicular magnetization i.e. it has easy-axis along z-axis. If K_U_Eff_ < 0, the magnet has easy-axis along the major-axis of the elliptical FL i.e. x-axis in this work. Among the parameters in equation (2) and equation (8), t_FL_ and N_z_ depend on the physical dimensions and remain fixed once the MTJ is fabricated, while E_z_ can be modified by controlling V_MTJ_. For precession based VCMA switching of the pMTJ devices^18,20^, these physical and electrical controls are designed such that in the absence of V_MTJ_, K_U_ which equals K_U_Bulk_ + K_I0_/t_FL_ is large enough for K_U_Eff_ to be positive. Howbeit, V_MTJ_ is designed to have a sufficient E_z_ that can render K_U_Eff_ to a computationally negative value. Physically, this implies that the FL destabilizes along z-axis and its easy-axis now aligns along x-axis, thereby forcing **m** to tend towards the new stable state. Conversely, if a negative voltage pulse is applied, as evident from equation (2) and equation (8), K_U_Eff_ becomes more strongly positive because the interface anisotropy is enhanced. Categorically, this has been suggested as a scheme to read MTJ with increased reliability^51^. V_C_ and E_zC_, respectively, symbolize the critical values, at which K_U_Eff_ is zero and thus the easy-axis orientation changes, of V_MTJ_ and E_z_ for given t_FL_. Moreover, E_z_ should not exceed the dielectric breakdown field E_Break_ which is slightly over 2 V-nm^−1^ ^10^, i.e. E_zC_ < V_0_/t_MgO_ < E_Break_. The design is furthermore constrained by a maximum permissible voltage in the system which is not discussed in this study because it is subjective to the targeted application and the desired stability factor Δ of FL. Δ is computed in the absence of V_MTJ_ and described as^52^,

9 $$\begin{array}{l}{\rm{\Delta }}={{\rm{\Delta }}}_{{\rm{0}}}{(1-\frac{{{\rm{H}}}_{{\rm{x}}}}{{{\rm{H}}}_{{\rm{K}}}^{{\rm{Eff}}}})}^{{\rm{2}}},\quad {{\rm{\Delta }}}_{{\rm{0}}}=\frac{{{\rm{K}}}_{{\rm{U}}\_{\rm{Eff}}}{\rm{V}}}{{{\rm{K}}}_{{\rm{B}}}{\rm{T}}},\quad {{\rm{H}}}_{{\rm{K}}}^{{\rm{Eff}}}=\frac{{{\rm{2K}}}_{{\rm{U}}\_{\rm{Eff}}}}{{{\mu }}_{{\rm{0}}}{{\rm{M}}}_{{\rm{S}}}}\cdot \end{array}$$

If the FL is permanently biased as in this work, the stability can be reduced quadratically, whereas if H_x_ is applied only during the write operation^53^, Δ can be substantially increased to equal Δ_0_. The scheme suggested in ref.^53^ depends on the Oersted field generated around the current carrying wire in the adjacent cells. Consequently, for a substantial H_x_ firstly it becomes power intensive and secondly this acts as a stray field and disturbs other bit-cells thereby limiting the memory density. Therefore, an alternative scheme of increasing Δ without compromising with V_MTJ_ or reducing H_x_ would be an important future direction.

**Figure 2:**
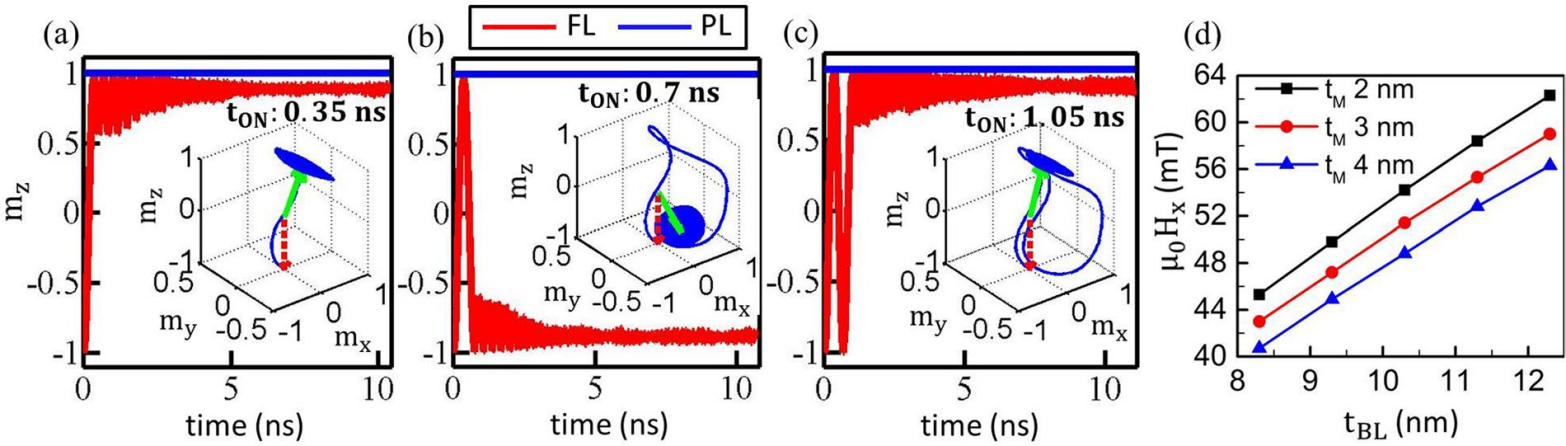
FL switching for a voltage pulse with t_ON_ in integer multiples of the half precession periods (t_Half_): t_ON_ is (**a**) 0.35 ns, (**b**) 0.7 ns and (**c**) 1.05 ns while t_Half_ is 0.35 ns. For the odd multiples the FL toggles while for the even cases it restores to the original state and remains unaltered. Insets show a full three dimensional (3D) FL dynamics for the respective cases, where the red dash arrow, green arrow and blue line is the initial **m** state, final **m** state and its trajectory, respectively. (**d**) Bias field acting on the FL along x-axis generated by the BL for different metal spacer thicknesses t_M_ and BL thicknesses t_BL_. This large enough exchange field, e.g. 50 mT for t_BL_ of 10 nm and t_M_ of 3 nm, can bias the FL for precession based VCMA switching.

For precession based VCMA switching, a V_MTJ_ as a trapezoidal pulse of duration t_Pulse_ is applied to toggle the FL, as shown in Fig. 1(b). A finite rise and fall time, t_Rise_ and t_Fall_, respectively, of 50 ps is assumed to consider a non-ideal input. A full-scale voltage V_0_ is applied for time t_High_ duration to temporarily change easy-axis from z- to x-axis. This induces the precession of **m** around the shifted **H**_**Eff**_. A sufficiently large H_x_ allows the FL m_z_ to swing from +1 to −1 and vice versa as illustrated in Fig. 2(a)–(c) for the FL of thickness t_FL_ 1.7 nm, cross-section 150 × 50 nm^2^, t_MgO_ of 2 nm, V_0_ of 1.6 V and *μ*_0_H_x_ of 50 mT. As shown in Fig. 2(a)–(c), by designing t_ON_ to be odd or even multiples of the half-precession period (t_Half_ = 0.35 ns for the shown cases), m_z_ toggles or comes back to the original state, respectively. A large enough t_OFF_ ensures that **m** relaxes to z-axis. Consequently, the final state of **m** strongly depends on t_ON_ since it determines if **m** is above or below the x-y plane when V_MTJ_ goes to zero and easy-axis is switched back along z-axis. This implies that pulse duration should be controlled in a certain range for deterministic switching.

Pessimistically, the deterministic switching based on precise control of precession cycles requires a bias magnetic field. To simplify the design of generating H_x_, an elliptical pMTJ is used, which allows an in-plane magnetized BL separated by a metal spacer to be fabricated within the MTJ stack (c.f. Fig. 1a). The dipole field provided by the BL is shown in Fig. 2(d). It shows that, typically as expected, a thinner metal spacer results in a stronger bias field because a dipole field strengthens as the distance from the magnet decreases. Intuitively, thickening the BL, whereas ensuring that a single domain is maintained, would have more magnetic moments along the x-axis, which then would lead to a larger bias field for the same t_M_. Hence, the t_M_ and the t_BL_ can be custom designed to obtain the required H_x_. In this work, the *μ*_0_H_x_ applied on the MTJ is approximately 50 mT. As shown in Fig. 2(d), this large H_x_ can be provided via a 10 nm thick BL with the metal spacer of t_M_ 3 nm, implying that an elliptical MTJ stack can function without an additional external system to provide the bias field.

### V_MTJ_ Dependence

First we investigate the electronic control of the device. To probe the effects of V_0_ on the precession based VCMA switching in an elliptical pMTJ, a representative FL of size 150 × 50 × 1.7 nm^3^ is chosen. The MTJ has t_MgO_ of 2 nm, resistance-area (RA) product of 1820 Ω-*μ*m^2^ and TMR_0_ of 144%^20^. A 50 mT bias field along x-axis H_x_ is applied to assist the switching. This in-plane field reduces the FL thermal stability from 138 to 28 as calculated from equation (9). However, because of the ultra-low power sub-nanosecond writing in this design, and the stability which suffices the requirements for embedded non-volatile cache memory^51,54,55^, the promulgated precessional VCMA based MRAM may be a promising replacement for power-intensive volatile static random-access memory (SRAM) in the cache. Furthermore, this stability also suffices for the MTJ based non-volatile logic (NVL)^56^.

**Figure 3:**
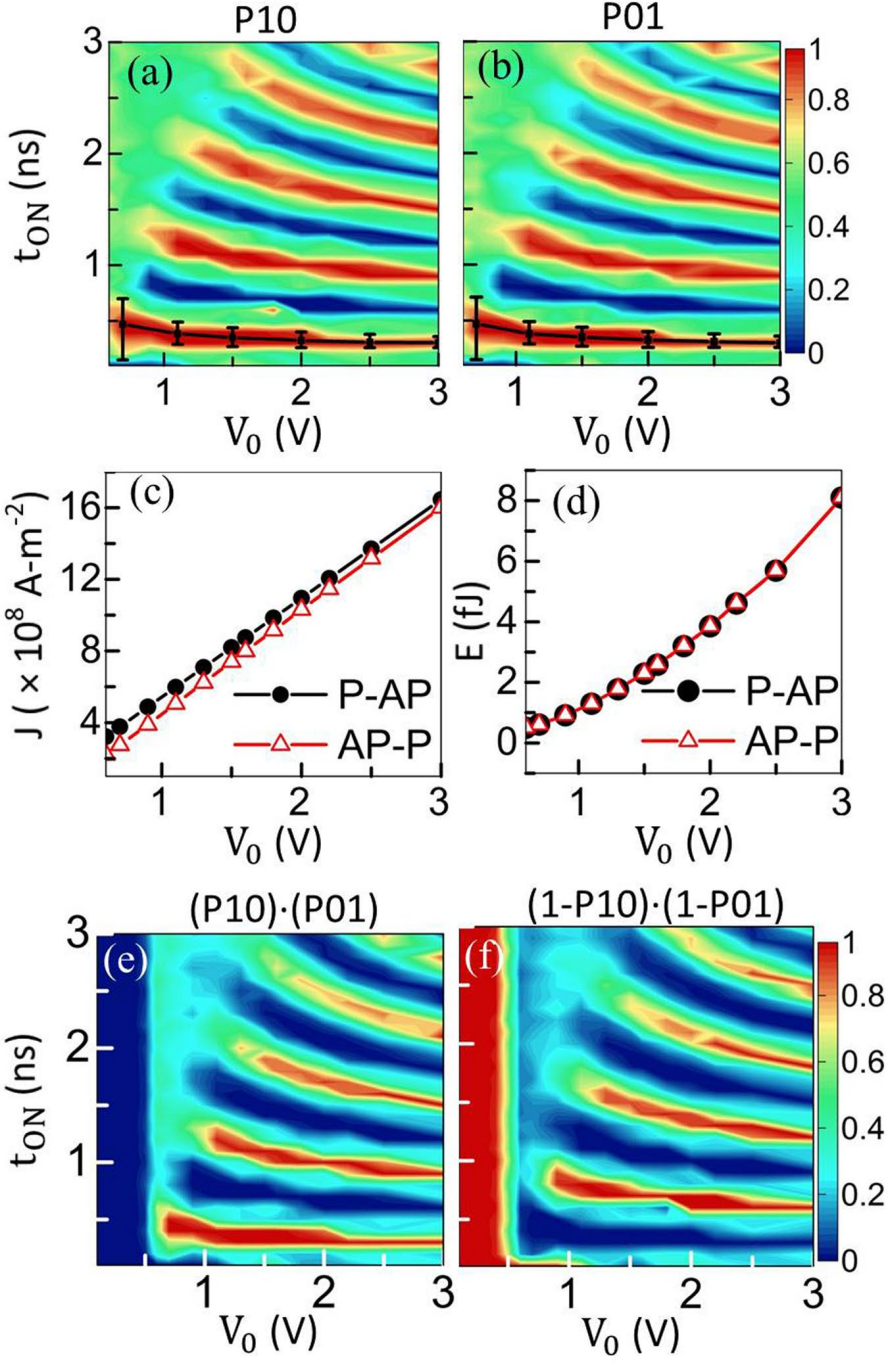
Phase diagram of switching probability from (**a**) parallel (P) to anti-parallel (AP) state (P10) and from (**b**) AP-to-P (P01) as a function of t_ON_ and V_0_ at t_MgO_ of 2 nm and *μ*_0_H_x_ of 50 mT (with thermal fluctuation). The black bars show the t _ON_ operation windows without considering thermal fluctuation. The increasing operation window as V_0_ decreases indicates that the device favors a low-voltage operation. (**c**) Current density and (**d**) energy consumption per switch *vs*. V_0_. (**e**) Switching probability from P-to-AP ‘AND’ AP-to-P (P10 ⋅ P01) as a function of t _ON_ and V_0_. Red regions indicate deterministic switching, i.e. 100% certainty of toggling. (**f**) Retention probability, i.e. (1-P10) ⋅ (1-P01) as a function of t_ON_ and V_0_. Red regions show with 100% probability that pre-configured data would not be altered.

The phase diagrams of the switching probability for switching from P-to-AP (P10) and from AP-to-P (P01) are shown in Fig. 3(a) and (b), respectively. For sweeping t_ON_, t_High_ is swept while t_Rise_ and t_Fall_ are held constant at 50 ps each. Each colour point is determined by simulating the device 100 times under the identical conditions while considering thermal fluctuation. Red regions (operation windows), which are directly related to the precession period, signify a deterministic toggling, i.e. 100% probability of switching, while the dark-blue regions denote an unaltered **m** state i.e. 100% probability that the original magnetic-state is retained. For given V_0_, the probability oscillates between 0 (the dark-blue regions) and 1 (the red regions) with t_ON_ because for the odd and even multiples of t_Half_, the FL toggles and gets restored to the original state, respectively. However, at small V_0_ and large t_ON_, the switching is nondeterministic and the probability is approximately 50%, as also observed experimentally in ref.^13^. This is because at low V_0_ and large t_ON_, the VCMA effect is relatively weak and t_High_ is comparable with the relaxation time, thus failing to keep the precession around x-axis for a long time and resulting in an uncertain final state in the presence of thermal fluctuation. Comparing Fig. 3(a) with 3(b) shows that there is no significant difference in the phase diagram for P10 and P01, which implies that there is symmetry in the switching from P-to-AP and AP-to-P, indicating that the STT effect, which always favours AP state in this work, is negligible. Next, the black curve with bars indicates deterministic switching without thermal fluctuation to explicitly illustrate the effect of thermal fluctuations for the fastest t_ON_ scenario. This t_ON_ equals t_Half_. As shown in Fig. 3(a) and (b), the operation window shrinks, as expected, when thermal fluctuation is considered, implying that for precession based VCMA switching it perturbs the deterministic toggling. Moreover, the operation window expands as V_0_ diminishes. This is because the reduced V_0_ weakens the VCMA effect, resulting in a stronger interfacial anisotropy field along z-axis as evident from equation (2). This tends to increase m_z_ and reduce m_x_ thus subduing shape anisotropy field along x-axis, resulting in the reduction of the x-component of **H**_**Eff**_. The **m** still precesses around x-axis, although its trajectory is now not totally symmetrical about the x-y plane. Because the precessional period is inversely proportional to the magnetic field along the precession-axis, which is nearly along x-axis, with a reduced field along x-axis the precession period increases. As a result, the operation window, which is closely related to the precession period, increases as V_0_ decreases. A larger operation window implies more tolerance in variation for t_High_, and hence, a more reliable write-operation, indicating that the low-voltage operation is achievable for this studied device.

The V_0_ dependence of current density *J* through the MTJ and energy consumption *E* is shown in Fig. 3(c) and (d), respectively, for both P-to-AP and AP-to-P switching of a unit probability with t_ON_ equal to a respective t_Half_. As expected, *J* increases linearly as V_0_ increases, and remains in the order of 10^8^ A-m^−2^ because of a thick enough (2 nm) MgO layer. This low current density then ensures a relatively low switching energy as seen in Fig. 3(d). The switching energy has two components,

10 $$\begin{array}{l}{\rm{E}}={\int }_{{\rm{0}}}^{{{\rm{t}}}_{{\rm{ON}}}}\frac{{({{\rm{V}}}_{{\rm{MTJ}}}({\rm{t}}))}^{{\rm{2}}}}{{{\rm{R}}}_{{\rm{MTJ}}}({\rm{t}})}{\rm{dt}}+\frac{{\rm{1}}}{{\rm{2}}}\frac{{{\rm{\varepsilon }}}_{{\rm{0}}}{{\rm{\varepsilon }}}_{{\rm{MgO}}}{\rm{A}}}{{{\rm{t}}}_{{\rm{MgO}}}}{{\rm{V}}}_{{\rm{0}}}^{{\rm{2}}},\end{array}$$

where ε_MgO_ = 9.7^57^ is the relative permittivity of MgO, ε_0_ is the vacuum permittivity, and *A* is the MTJ cross-sectional area. The first term in equation (10) is the Joule heating E_J_, and second term is the charging energy E_C_ consumed by the MTJ capacitance. The capacitance has been assumed to be independent of the relative magnetization of PL and FL because of the sub-*μ*m^2^ MTJ cross-section^58^. E_C_ ranges from 0.046 fJ to 1.138 fJ for V_0_ from 0.6 V to 3 V, which is 9% to 14% of *E* (0.5 fJ to 8.1 fJ), respectively. Because the current density stays lower than 2 × 10^9^ A-m^−2^, a low switching energy (<10 fJ/switch) is achieved (c.f. Table I of ref.^56^. for a general comparison with other non-volatile memory technologies). Moreover, Fig. 3(e) exhibits the switching probability from P-to-AP ‘AND’ AP-to-P as a function of t_ON_ and V_0_. Red regions denote a deterministic switching, i.e. 100% certainty of toggling. At V_0_ = 1 V, the corresponding operation window of t_ON_ is from 0.31 ns to 0.46 ns i.e. 0.15 ns, a decent margin for t_High_ to vary without affecting the reliable operation. On the other hand, Fig. 3(f) presents the retention probability as a function of t_ON_ and V_0_. Red regions show with 100% probability that pre-configured data is not disturbed. As a consequence, the red regions can be used for reading, e.g. as long as the read voltage is below 0.4 V, the FL magnetic state will always remain unaltered, which implies that an absolutely disturb-free read operation can be achieved for the MRAM application.

### MgO Thickness Dependence

**Figure 4:**
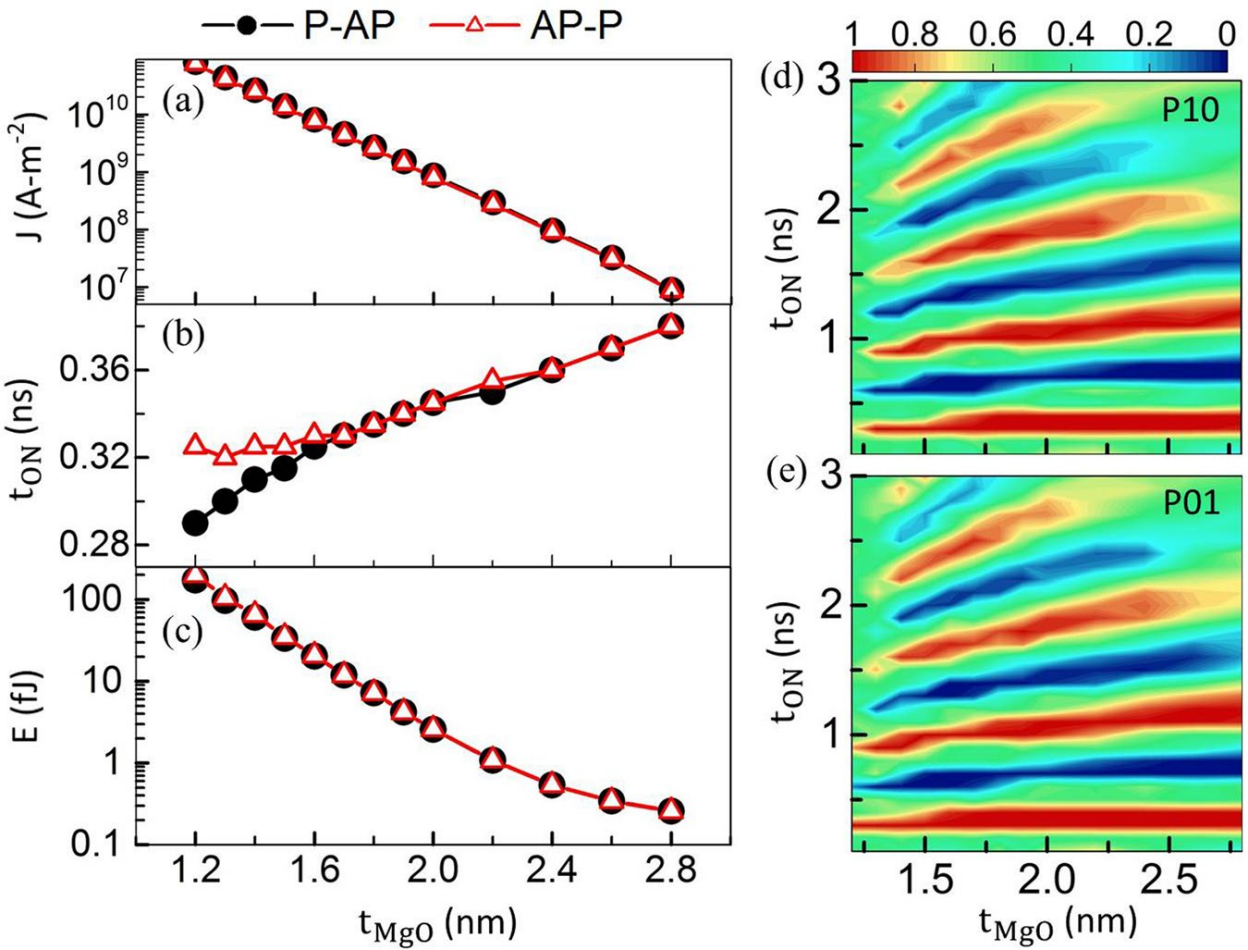
Effects of t_MgO_ on (**a**) current density, (**b**) t_ON_ (optimal values used to switch, i.e. t_Half_) and (**c**) energy consumption per switching operation at 1.6 V V_0_ and 50 mT *μ*_0_H_x_. Red lines with open triangles show the process from AP-to-P. Black lines with solid circles correspond to the process from P-to-AP. The smallest switching energy achieved is 0.3 fJ. Phase diagram for switching probability from (**d**) P-to-AP and (**e**) AP-to-P *vs*. t_ON_ and t_MgO_.

As demonstrated in the previous section, the device prefers a low-voltage operation for a decent pulse duration margin unto a value at which further decreasing V_0_ may fail the switching. Hence, to study the t_MgO_ dependence on the switching, V_0_ is fixed to be 1.6 V in this section. This V_0_ allows a sufficient t_MgO_ variation range to achieve low energy consumption as expounded later. RA-products, TMRs and V_Half_ for different MgO thicknesses are extracted from the experimental paper^20^. For a fixed V_0_ as the t_MgO_ decreases, principally, both VCMA and STT effect become stronger. The former becomes stronger because E_z_ at the MgO-FL interface becomes stronger (see equation (2)). Simultaneously, the RA-product decreases exponentially as t_MgO_ decreases. Since *J* is inversely proportional to the RA-product, *J* thus increases exponentially from 10^7^ A-m^−2^ to 10^10^ A-m^−2^ for decreasing t_MgO_, as shown in Fig. 4(a), which makes the STT increasingly stronger. Figure 4(b) shows that at large t_MgO_, the two curves of t_ON_, which is chosen to equal to t_Half_, overlap, indicating that the VCMA effect dominates over the STT effect. Besides, a linear relation is observed on account of the fact that t_ON_ is inversely proportional to the precession frequency which in turn is almost proportional to **H**_**Eff**_. The **H**_**Eff**_ varies linearly with **H**_**K**_, and **H**_**K**_ is inversely proportional to t_MgO_ (see equation (2)) because of the VCMA effect. For small t_MgO_, there is a divergence in t_ON_ between P-to-AP (black solid circles) and AP-to-P (red triangles) switching trends, implying that STT effect is substantial which can be understood as follows. Beyond the critical electric field which changes the easy axis from z- to x-axis, further strengthening of VCMA effect is inessential and has no significant additional contribution in the switching. However, STT effect has no such upper threshold in this case and starts to dictate the switching dynamics. Since the electron flow direction is from FL to PL, for P-to-AP switching, due to STT effect, the FL receives spin-flux anti-parallel to **M**_**PL**_. As a result, STT effect assists VCMA effect to attain an AP state and accelerates the switching process. In contrast, for AP-to-P switching, STT effect still endeavors to maintain the FL in the AP state while VCMA effect strives to toggle the FL into a P-state. The two effects thus jostle to toggle the FL. This decelerates the toggling, which results in a larger t_ON_.

As discussed in the previous section, E_C_ is a fraction of the Joule heating. When STT effect is substantial, the *E* shown in Fig. 4(c) nearly follows the declining trend of *J* in Fig. 4(a) since the switching time variation is relatively small. Nevertheless, when t_MgO_ becomes thicker than 2.2 nm, E_C_ becomes comparable to E_J_ because of the exponential decline in *J* and the corresponding Joule heating. Thus, for large t_MgO_, VCMA effect dominates, and the *E* slope tapers down with *E* approaching 0.3 fJ, which is also the minimum energy achieved in this work. Figure 4(d) and (e), respectively, show the phase diagrams of switching probability from P-to-AP (P10) and from AP-to-P (P01) as a function of t_ON_ and t_MgO_. An obvious oscillatory dependence on t_ON_ is observed. At large t_MgO_ and for long pulse duration, the oscillations disappear because of weak VCMA effect. Interestingly in Fig. 4(d), there is a sharp decrease in the t_ON_ operation window for the first half precession cycle for small t_MgO_ (the red region on the left-bottom around t_MgO_ = 1.6 nm). This happens because in the said region a strong STT effect compliments the VCMA effect for P-to-AP switching, and greatly accelerates the switching process. This sharply reduces the precession period and the scope for tolerating variations in t_High_. For larger t_MgO_, a wider operation window indicates that the device can tolerate more variations in t_High_. It can be found that it is more favorable to design the device with a large MgO thickness for given V_0_, because both a larger margin in the pulse variation for deterministic switching and a lower write-energy can be achieved.

### MTJ Scalability and Bias Magnetic Field

As noted earlier, the external bias field required in the switching has been a critical bottleneck in advancing it for memory applications and therefore in this work, the elliptical pMTJs have been presented so that H_x_ can be engineered within the stack and provided by a BL. Besides the thickness of BL discussed earlier, it is the MTJ cross-section that determines the number of magnetic moments in the BL to provide a bias field through the FL. Furthermore, the demagnetizing field scales with the cross-section thereby modifying the switching time, required bias field and energy landscape. Therefore, the MTJ cross-section is an important physical constraint to investigate and comprehend the device physics in our proposed VCMA device.

**Figure 5:**
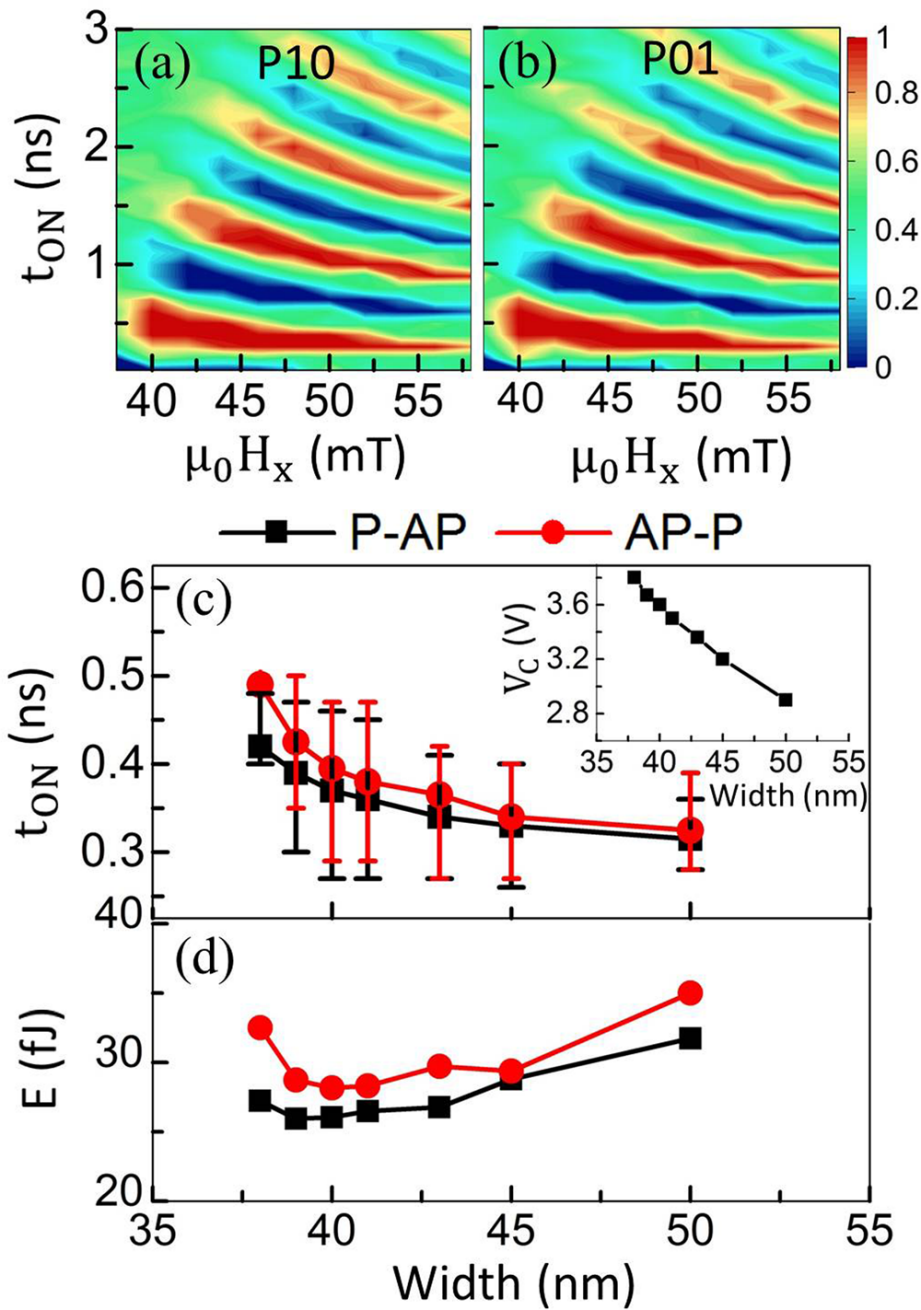
Phase diagram for switching probability of (**a**) P-to-AP and (**b**) AP-to-P as a function of t_ON_ and bias magnetic field *μ*_0_H_x_. H_x_ is the magnitude of **H**_**Bias**_ projecting along x-axis. Optimal t_ON_ used to switch (t_Half_) in (**c**) and energy consumption in (**d**) as a function of the MTJ width. The AR and FL thickness are held constant at 3 and 1.7 nm respectively, e.g. for width of 40 nm, the MTJ cross-section is 40 × 120 nm^2^.The black and red bars indicate the t_ON_ operation regions during which switching probability is 100%. The inset shows the critical voltage V_C_ *vs*. the MTJ width.

The effect of H_x_ on the switching probability is shown in Fig. 5(a) and (b). For a pMTJ with FL of 150 × 50 × 1.7 nm^3^ dimensions, t_MgO_ of 2 nm and V_0_ of 1.6 V, there is a limited functional region in the range of 38–58 mT for *μ*_0_H_x_. This range can be shifted for different conditions. As seen from Fig. 5(a) and (b), increasing the bias field H_x_ shrinks the red region, i.e. the operation window, which is similar to the case exhibited in Fig. 3(a) and (b). When *μ*_0_H_x_ increases to more than 58 mT, an excessively strong **H**_**Eff**_ results in overly fastened precession, thus sharply reducing the relaxation time, which is too fast to allow deterministic switching. Conversely, if *μ*_0_H_x_ is insufficient, i.e. less than 38 mT, deterministic switching around x-axis would not be supported. Hence, a probabilistic final state is attained by virtue of thermal fluctuation. To reduce the required H_x_ for switching, one possible way is to design t_FL_ even closer to the critical thickness but this would further sacrifice Δ. This adjustment has three effects: it would weaken both the interfacial anisotropy and the demagnetizing field along z-axis, and enhance the demagnetizing field along x-axis. All of these would enable the operation at smaller V_C_. In consequence, a stronger VCMA effect is obtained at the same V_0_, which then relieves the requirement for larger *μ*_0_H_x_.

Scalability of the pMTJ is next investigated in Fig. 5(c) and (d). The AR is held at 3, t_FL_ at 1.7 nm, t_MgO_ at 1.5 nm and V_0_ at 1.6 V, while the MTJ length and width are swept. The bars in Fig. 5(c) represent the operation windows, within which switching happens with 100% certainty. Figure 5(c) also shows that when the MTJ cross-section (represented as MTJ width) is scaled down, the optimal t_ON_ (the data-markers on the curve), which equals respective t_Half_, increases. This happens because on scaling down the MTJ, N_z_ decreases which thus increases K_U_Eff_. As a result, V_C_ (c.f. the inset), where K_U_Eff_ = 0, as seen from equation (2) and equation (8), becomes larger. In consequence, it is more difficult to switch. Hence, it takes larger t_ON_ or the switching may even fail altogether. It is found that a considerable operation window is achieved for the MTJ cross-section between 39 × 117 nm^2^ and 45 × 135 nm^2^.

For the designs in Fig. 5(c), the respective energy consumption is shown in Fig. 5(d). At first a descending and then an ascending trend is observed when scaling down on account of the competition between t_ON_ and the MTJ resistance as evinced in equation (10). The former increases as observed in Fig. 5(c), while the latter also increases because for given RA-product, the MTJ resistance increases as the MTJ cross-section reduces. These two have opposite contributions to the Joule heating; therefore, the trends exhibit a local minima. These trends also imply that unduly scaling down the MTJ cross-section may not be attractive in terms of energy consumption.

## Conclusion

We propose and appraise the elliptical pMTJs for voltage controlled precessional switching. The V_MTJ_, t_MgO_, STT, bias magnetic field and MTJ scalability effects on the pMTJ properties are investigated. We show that an in-plane magnetized BL designed within the MTJ stack can bias the FL to eliminate the need of providing a uniform in-plane magnetic field for the FL by an additional circuit. The pMTJ can be switched for as low as 0.3 fJ in just 0.38 ns at 1.6 V. Furthermore, it is shown that Joule heating can be adequately suppressed by increasing t_MgO_. We also find that the design favors to operate at low voltage (~1 V) and large MgO thickness. There is also a sufficient margin for the variation in t_High_ without affecting the reliable operation. This should be encouraging for a practical disposition of the VCMA based MRAM. The advantages like fast switching, ultra-low energy consumption and non-volatility are very attractive for VCMA based MRAM application in cache memories.
